# A Possible World-Based Fusion Estimation Model for Uncertain Data Clustering in WBNs

**DOI:** 10.3390/s21030875

**Published:** 2021-01-28

**Authors:** Chao Li, Zhenjiang Zhang, Wei Wei, Han-Chieh Chao, Xuejun Liu

**Affiliations:** 1 Department of Electronic and Information Engineering, Key Laboratory of Communication and Information Systems, Beijing Municipal Commission of Education, Beijing Jiaotong University, Beijing 100044, China; 15111037@bjtu.edu.cn; 2 The School of Software Engineering, Beijing Jiaotong University, Beijing 100044, China; 3 Shaanxi Key Laboratory for Network Computing and Security Technology, School of Computer Science and Engineering, Xi’an University of Technology, Xi’an 710048, China; weiwei@xaut.edu.cn; 4 Department of Electrical Engineering, National Dong Hwa University, Hualien 97401, Taiwan; hcc@mail.ndhu.edu.tw; 5 School of Information Engineering, Beijing Institute of Petrochemical Technology, Beijing 102617, China; lxj@bipt.edu.cn

**Keywords:** possible worlds, fusion estimation, uncertain data, clustering

## Abstract

In data clustering, the measured data are usually regarded as uncertain data. As a probability-based clustering technique, possible world can easily cluster the uncertain data. However, the method of possible world needs to satisfy two conditions: determine the data of different possible worlds and determine the corresponding probability of occurrence. The existing methods mostly make multiple measurements and treat each measurement as deterministic data of a possible world. In this paper, a possible world-based fusion estimation model is proposed, which changes the deterministic data into probability distribution according to the estimation algorithm, and the corresponding probability can be confirmed naturally. Further, in the clustering stage, the Kullback–Leibler divergence is introduced to describe the relationships of probability distributions among different possible worlds. Then, an application in wearable body networks (WBNs) is given, and some interesting conclusions are shown. Finally, simulations show better performance when the relationships between features in measured data are more complex.

## 1. Introduction

Clustering is a kind of technology for machine learning that puts similar objects into the same cluster. Clustering techniques play an important role in many areas such as health care and action recognition in the medical domain [1,2], behavior surveillance and battlefield prediction in the military field [3,4], resource and information management in the communications field [5,6], and so on. There are plenty of cluster methods presented that can be divided into three principal types according to the clustering scale: distance-based, density-based, and connectivity-based [7,8].

Most clustering methods focus on deterministic data. Unfortunately, almost all clustering data are collected by the corresponding equipment, which entails measuring errors. In this case, the uncertain data can describe the measurement data better. For acquiring better and more appropriate results, the fusion estimation methods such as the Bayes-based [9], Kalman-based [10], or artificial intelligence-based [11,12] methods are commonly used to estimate the measurements.

Fusion estimation is a technology that uses the computing power of data acquisition equipment to de-noise and de-redundancy the measurement data according to certain rules. It focuses on mining data information, designing corresponding estimation algorithms, and improving the accuracy of data. In this technology, the measurement data are de-noised first, and then the data is fused on the time series to obtain the accurate conclusion for uncertain data. Finally, the uncertain data is processed by clustering, and the final processing result is obtained.

Many methods have been proposed to deal with uncertain data in recently years [13,14,15]. Among these methods, the possible world-based methods have been demonstrated to be efficient and reasonable. Possible world-based clustering methods consider all the probabilities of the uncertain data and fuse them into the final clustering result. This kind of method usually exhibits good performance. On the other hand, the uncertain data can be represented by a probability distribution in most cases. Therefore, the Kullback–Leibler divergence (KL divergence) [16] is used to describe the similarity of two probability distributions.

In practice, there are differences in the accuracy of different acquisition equipment, which is represented by the differences in data uncertainty. Existing algorithms based on possible worlds can deal with the difference problem of uncertainty in a relatively simple way. In this paper, variance, an important statistic of data uncertainty, is introduced into the model of possible worlds to study its role in improving accuracy. Then, a possible world-based fusion estimation model (PWFEM) for uncertain data is presented, which includes two methods according to different distance-based formulas. When the variance of uncertain data is small, the numerical distance-based method (PWFEM-nd) is employed. The probabilistic distance-based method (PWFEM-pd) is employed when variance is prominent. Then, the application in wearable body networks (WBNs) is introduced. The specific derivation formula is given with the different distance-based formulas. Finally, the simulations show good performance in terms of the proposed model.

The rest of the paper is organized as follows. In Section 2, the related works are introduced. In Section 3, the preliminaries are introduced, and some definitions and assumptions are given. The theoretical derivation of the PWFEM is given in Section 4. In Section 5, the simulations examine the performance of the PWFEM. Finally, conclusions are given in Section 6.

## 2. Related Works

In this section, the processing technologies of uncertain data are introduced in detail. The collected data that come from acquisition equipment contain noise, which means the collected data contain great uncertainty. Therefore, it is necessary to perform fusion estimation processing on the data first, and use the rules and redundancy of the data itself to improve the data accuracy and reduce the uncertainty of the data.

Commonly used fusion estimation algorithms include Bayes filter (BF) [17], Kalman filter (KF) [18], extended Kalman filter (EKF) [19], unscented Kalman filter (UKF) [20], and particle filter (PF) [21]. Wherein, BF and KF are estimates of linear systems, BF can theoretically estimate data of arbitrary noise distribution, and KF is BF when the noise is Gaussian white noise. The EKF, UKF, and PF are the estimates of the nonlinear system, where EKF is for weak nonlinear system, UKF is for strong nonlinear systems and has high computing complexity, while the PF is calculated directly from the average probability density conditions, in which the probability density is determined by EKF and UKF approximation, but the estimation precision is higher than that of a single use of EKF or UKF, but the number of calculations is much higher than that of EKF and UKF.

In [22], the authors argued that two possible world-based clustering algorithms suffered from the following issues: (1) they dealt with each possible world independently and ignored the consistency principle across different possible worlds; (2) they required an extra post-processing procedure to obtain the results, which meant that effectiveness was highly dependent on the post-processing method, and their efficiencies were also not very good. In order to solve the problems above, Liu et al. proposed a possible world-based consistency learning model that considered the consistency principle during the clustering/classification procedure and thus could achieve satisfactory performance.

The Possible world based consistency learning model for clustering uncertain data (PWCLU) was proposed in [22], which holds that the clustering results in each possible world are consistent. Several equipment types were used for collecting the same data. Each piece of data for one piece of equipment was considered to belong to a possible world, and the probability was regarded as equal for each possible world. The authors only gave an algorithm to deal with finite possible worlds.

On the other hand, clustering algorithms usually require a method to describe the distance between two datasets. In uncertain data, the distance can be expressed as a probability distribution in most cases. Therefore, a method of describing the distance between probability distributions is required. Sinkkonen and Kaski [23] studied the problem of learning groups or categories that were local in the continuous primary space but homogeneous according to the distributions of an associated auxiliary random variable over a discrete auxiliary space. In their model, Kullback–Leibler divergence was used to calculate the distance between two probability distributions.

In this paper, a possible world-based fusion estimation model (PWFEM) is proposed for clustering uncertain data. The proposed model removes the assumption of the consistency principle of [22]. Moreover, two PWFEM-based methods are given. One generalizes the PWCLU to the continuous possible worlds, which is based on numerical distance. Therefore, it is called PWFEM-nd. The other is based on probability distribution distance and is named PWFEM-pd. Then, an application in WSNs is discussed. Two specific distance functions that correspond to the numerical distance and probability distribution distance, respectively, are introduced to prove that the PWFEM-nd is equivalent to PWFEM-pd under certain circumstances. Finally, the simulations are discussed; they showed good performance of the models.

## 3. Preliminaries

In this section, some necessary definitions and assumptions are given for possible world and Kullback–Leibler divergence; the assumptions of independence for each component of the datasets and the structure of the data are also given. 

### 3.1. Definition of Possible World

Let $O\in R^{N\times n},O=\left\{O_{1},O_{2},\cdots ,O_{n}\right\}$ be an uncertain dataset, where *O* is not deterministic data but a probability distribution. If *O* is a discrete probability distribution, *pw* is one of the possibilities of the uncertain data *O*, which can be written as $pw=\left\{O_{1}^{pw},O_{2}^{pw},\cdots ,O_{n}^{pw}\right\}$, which is deterministic data with its probability *P*(*pw*). If *O* is a continuous probability distribution, *O* can be described as a probability density function *f*(*pw*), where *pw* is the value of the random variable *O*. Then,

$$\int_{D}f\left(pw\right)dpw=1$$

### 3.2. Definition of Kullback–Leibler Divergence

Let *p*(*x*) and *q*(*x*) be the distribution of random variable *X*, so the Kullback–Leibler divergence of *p*(*x*) and *q*(*x*) is:

(1) $$d_{KL}\left(p\left(x\right),q\left(x\right)\right)=\int_{-\infty }^{+\infty }p\left(x\right)\log\left(\frac{p\left(x\right)}{q\left(x\right)}\right)dx$$

### 3.3. Some Assumptions

**Assumption 1.** Almost all possible worlds exhibit the same class labels and cluster structures, and they exhibit the different class labels and cluster structures with small probabilities.

**Assumption 2.** In Section 5, it is assumed that ∀*x_i_*, *x_j_*∈*X*, *x_i_* + *x_j_* is also the Gaussian distribution.

**Assumption 3.** In Section 5, it is assumed that the wearable nodes keep a stable state to collect the data all the time. Therefore, the covariance matrix will not change.

## 4. Possible World-Based Fusion Estimation Model (PWFWM)

In this section, the details of the PWFWM are introduced in three parts. The first part is the introduction of data fusion estimation. The second part is the introduction of the calculation process of distribution distance. The third part introduces the clustering method based on the possible world.

### 4.1. Data Fusion Estimation

The collected data can be divided into two types: filterable data and high accuracy data. Without loss of generality, it is assumed the measurement data at time *t* is:

(2) $$M_{t}={\left[z_{1}^{f},z_{2}^{f},\cdots ,z_{q}^{f},z_{1}^{a},z_{2}^{a},\cdots ,z_{s}^{a}\right]}_{t}$$

where $M_{t}^{f}={\left[z_{1}^{f},z_{2}^{f},\cdots ,z_{q}^{f}\right]}_{t}$ are the filterable data, and $M_{t}^{a}={\left[z_{1}^{a},z_{2}^{a},\cdots ,z_{s}^{a}\right]}_{t}$ are the high-accuracy data.

Corresponding to the possible world, filterable data are the probabilistic data, while the high accuracy data are the numeric data. It is assumed the format of the clustering data in a possible world at time *t* is:

(3) $$X_{t}={\left[x_{1}^{p},x_{2}^{p},\cdots ,x_{h}^{p},x_{1}^{n},x_{2}^{n},\cdots ,x_{s}^{n}\right]}_{t}$$

where $X_{t}^{p}={\left[x_{1}^{p},x_{2}^{p},\cdots ,x_{h}^{p}\right]}_{t}$ are the probability data, and $X_{t}^{n}={\left[x_{1}^{n},x_{2}^{n},\cdots ,x_{s}^{n}\right]}_{t}$ are the numeric data.

In most cases, the filterable data can be obtained according to the Kalman-based filter. The high accuracy data can be converted to filterable data by the Gaussian distribution, whose expectation is zero and whose variance is small. The details are as follows.

The measurement data are first converted to the clustering data by the following formulas:

If the filterable data satisfied the following state function and measurement function:

(4) $$\left\{ \begin{array}{l} X_{t}^{p}=f\left(X_{t-1}^{p}\right)+\omega_{t/t-1}\\ M_{t}^{f}=g\left(X_{t-1}^{p}\right)+\upsilon_{t} \end{array}\right.$$

The appropriate filter algorithm can be used to solve the functions above. If the result is ${\hat{X}}_{t}^{p}$, the probability data can be written as ${\hat{X}}_{t}^{p}+\omega_{t/t-1}$. 

Similarly, the numerical data can be written as $X_{t}^{n}=M_{t}^{a}+\omega_{t}^{a}$, where $\omega_{t}^{a}$ is Gaussian distribution with zero mean and small variance. 

Then, we have:

(5) $$X_{t}=\left[\begin{array}{l} {\hat{X}}_{t}^{p}+\omega_{t/t-1}\\ M_{t}^{a}+\omega_{t}^{a} \end{array}\right]=\left[\begin{array}{l} {\hat{X}}_{t}^{p}\\ M_{t}^{a} \end{array}\right]+\left[\begin{array}{l} \omega_{t/t-1}\\ \omega_{t}^{a} \end{array}\right]={\hat{X}}_{t}+\Omega_{t}$$

where ${\hat{X}}_{t}=\left[\begin{array}{l} {\hat{X}}_{t}^{p}\\ M_{t}^{a} \end{array}\right]$ and $\Omega_{t}=\left[\begin{array}{l} \omega_{t/t-1}\\ \omega_{t}^{a} \end{array}\right]$. Moreover, we let $\Omega_{t}=\left[\begin{array}{l} \omega^{p}\\ \omega^{a} \end{array}\right]$, which is a scleronomic Gaussian distribution. Therefore, according to Assumption 2, the multivariate Gaussian distribution with *X_t_* can be written as follows:

(6) $$X=\frac{1}{{\left(\sqrt{2\pi }\right)}^{l}{\left|\Sigma \right|}^{\frac{1}{2}}}e^{-\frac{{\left(x-\mu_{x}\right)}^{T}{\left(\Sigma \right)}^{-1}\left(x-\mu_{x}\right)}{2}}$$

where *l* = *h* + *s*, $\mu_{x}={\left[E\left(x_{i}\right)\right]}_{i=1}^{l}$ and

(7) $$\Sigma ={\left[\sigma_{ij}\right]}_{l\times l},\sigma_{ij}=\sqrt{D\left(x_{i}\right)\cdot D\left(x_{j}\right)}$$

Based on the above, the structure of clustering data can be confirmed. Then, the distance-based functions need to be confirmed.

### 4.2. Distance Calculation Method Based on KL Divergence-Based Distance 

Almost all clustering algorithms need to calculate the distance. In the PWFWM, there are two types of data: filterable and high accuracy. For accuracy data, the Euclidean distance can be used, and the KL divergence can be used to process the filterable data. In this Section, the distance calculation method based on KL divergence is introduced in detail.

KL divergence analyzes the degree of difference between two distributions from the perspective of information entropy. Assume that *p*(*x*) and *q*(*x*) are two distributions of random variable X, then the KL divergence is:

(8) $$KL\left(p∥q\right)=\int_{-\infty }^{+\infty }p\left(x\right)\log\frac{p\left(x\right)}{q\left(x\right)}dx$$

The calculation formula in the discrete case is:

(9) $$KL\left(p∥q\right)=\sum_{i=1}^{n}p\left(x_{i}\right)\log\frac{p\left(x_{i}\right)}{q\left(x_{i}\right)}$$

Assuming that the probability distribution is usually Gaussian, $P∼N\left(\mu_{1},\Sigma_{1}\right)$ and $Q∼N\left(\mu_{2},\Sigma_{2}\right)$, and the dimension of the data is *n*. Then, the KL divergence calculation formula is as follows:

(10) $$KL\left(P∥Q\right)=\int_{-\infty }^{+\infty }p\left(x\right)\log\frac{p\left(x\right)}{q\left(x\right)}dx=E_{p}\left[\log p\left(x\right)-\log q\left(x\right)\right]$$

Plugs the $P∼N\left(\mu_{1},\Sigma_{1}\right)$ and $Q∼N\left(\mu_{2},\Sigma_{2}\right)$ in (10):

(11) $$\begin{array}{ll} KL\left(P∥Q\right) & =\frac{1}{2}E_{P}\left[\log\frac{\left|\Sigma_{2}\right|}{\left|\Sigma_{1}\right|}-{\left(x-\mu_{1}\right)}^{T}\Sigma_{1}^{-1}\left(x-\mu_{1}\right)+{\left(x-\mu_{2}\right)}^{T}\Sigma_{2}^{-1}\left(x-\mu_{2}\right)\right]\\ & =\frac{1}{2}\log\frac{\left|\Sigma_{2}\right|}{\left|\Sigma_{1}\right|}-\frac{1}{2}E_{P}\left[{\left(x-\mu_{1}\right)}^{T}\Sigma_{1}^{-1}\left(x-\mu_{1}\right)\right]+\frac{1}{2}E_{P}\left[{\left(x-\mu_{2}\right)}^{T}\Sigma_{2}^{-1}\left(x-\mu_{2}\right)\right] \end{array}$$

where

(12) $$\begin{array}{l} E_{P}\left[{\left(x-\mu_{1}\right)}^{T}\Sigma_{1}^{-1}\left(x-\mu_{1}\right)\right]=E_{P}\left[tr\left(\Sigma_{1}^{-1}\left(x-\mu_{1}\right){\left(x-\mu_{1}\right)}^{T}\right)\right]\\ =tr\left[E_{P}\left(\Sigma_{1}^{-1}\left(x-\mu_{1}\right){\left(x-\mu_{1}\right)}^{T}\right)\right]=tr\left[\Sigma_{1}^{-1}E_{P}\left(\left(x-\mu_{1}\right){\left(x-\mu_{1}\right)}^{T}\right)\right]=n \end{array}$$

and

(13) $$\begin{array}{l} E_{P}\left[{\left(x-\mu_{2}\right)}^{T}\Sigma_{2}^{-1}\left(x-\mu_{2}\right)\right]=E_{P}\left[tr\left(\Sigma_{2}^{-1}\left(x-\mu_{2}\right){\left(x-\mu_{2}\right)}^{T}\right)\right]\\ =tr\left[\Sigma_{2}^{-1}E_{P}\left(\left(x-\mu_{2}\right){\left(x-\mu_{2}\right)}^{T}\right)\right]=tr\left[\Sigma_{2}^{-1}E_{P}\left(xx^{T}-x\mu_{2}^{T}-\mu_{2}x^{T}+\mu_{2}\mu_{2}^{T}\right)\right]\\ =tr\left[\Sigma_{2}^{-1}\left(\Sigma_{1}+\mu_{1}\mu_{1}^{T}-\mu_{1}\mu_{2}^{T}-\mu_{2}\mu_{1}^{T}+\mu_{2}\mu_{2}^{T}\right)\right]=tr\left[\Sigma_{2}^{-1}\Sigma_{1}+\Sigma_{2}^{-1}\left(\mu_{1}-\mu_{2}\right){\left(\mu_{1}-\mu_{2}\right)}^{T}\right]\\ =tr\left(\Sigma_{2}^{-1}\Sigma_{1}\right)+{\left(\mu_{1}-\mu_{2}\right)}^{T}\Sigma_{2}^{-1}\left(\mu_{1}-\mu_{2}\right) \end{array}$$

Finally, we have

(14) $$KL\left(P∥Q\right)=\frac{1}{2}\left[\log\frac{\left|\Sigma_{2}\right|}{\left|\Sigma_{1}\right|}-n+tr\left(\Sigma_{2}^{-1}\cdot \Sigma_{1}\right)+{\left(\mu_{1}-\mu_{2}\right)}^{T}\Sigma_{2}^{-1}\left(\mu_{1}-\mu_{2}\right)\right]$$

Moreover, if Σ_1_ = Σ_2_ = Σ. Then, we get:

(15) $$d_{KL}\left(i,j\right)=KL\left(P∥Q\right)=\frac{1}{2}{\left(u_{j}-u_{i}\right)}^{T}\Sigma^{-1}\left(u_{j}-u_{i}\right)$$

In this way, the distance between two probability distributions is obtained. Then, the clustering method based on the possible world can be used.

### 4.3. The Clustering Method Based on the Possible World

In [22], the authors used an adaptive, local-structure learning method to calculate the consensus affinity matrix. In their model, the collected numerical data are used to match the probability density function (PDF) of the uncertain data. However, the authors give no algorithm for the case where the PDF is given directly. Moreover, the proposed method needs a sizable quantity of data. In this paper, Assumption 1 is proposed instead of the consistency principle.

According to Assumption 1 above, the probability of each possible world should be considered when calculating the consensus affinity matrix. Then, the objective function is shown as follows:

(16) $$\begin{array}{l} \min\sum_{j=1}^{n}d_{ij}^{pw}s_{ij}^{pw}+\alpha \sum_{j=1}^{n}s_{ij}^{pw}\\ s.t.\;S_{i}^{pw}={\left[s_{1i}^{pw},s_{2i}^{pw},\cdots ,s_{ni}^{pw}\right]}^{T}\\ {\left(S_{i}^{pw}\right)}^{T}\cdot 1^{n\times 1}=1\\ 0\leq s_{ij}^{pw}\leq 1 \end{array}$$

where, $d_{ij}^{pw}$ is a kind of distance function between $O_{i}^{pw}$ and $O_{j}^{pw}$, and $S_{i}^{pw}={\left[s_{1i}^{pw},s_{2i}^{pw},\cdots ,s_{ni}^{pw}\right]}^{T}$ is the normalized distance matrix for one of the possible worlds (*pw*).

Moreover, let the effective results of *S_i_*

(17) $$t=\sum_{j=1}^{n}\mathrm{sgn}\left(s_{ij}^{pw}\right)$$

According to the conclusion of [22], *t* can be adjusted by *α*, and the optimization result is

(18) $$s_{ij}^{pw}=\frac{1}{t}+\frac{1}{2\alpha }\left(\frac{\sum_{s=1}^{t}d_{is}^{\prime pw}}{t}-d_{ij}^{pw}\right)$$

where $D_{i}^{\prime pw}={\left[d_{1i}^{\prime pw},d_{2i}^{\prime pw},\cdots ,d_{ni}^{\prime pw}\right]}^{T}$ is another order of $D_{i}^{pw}$, and it ranges from small to large.

According to the formulas above, the extra information about classes is required to confirm *t*. It is set as *t* = *N* if there is no extra information about classes. That is:

(19) $$s_{ij}^{pw}=\frac{1}{n}+\frac{1}{2\alpha }\left(\frac{\sum_{s=1}^{n}d_{is}^{\prime pw}}{n}-d_{ij}^{pw}\right)$$

Finally, an optimization normalized distance matrix S* is needed for clustering the training set, which is satisfied by the following optimal model:

(20) $$\begin{array}{l} \min E\left({\left\|S-S^{pw}\right\|}_{F}^{2}\right)\\ s.t.\;{\left(S_{i}\right)}^{T}\cdot 1^{n\times 1}=1\\ 0\leq s_{ij}\leq 1 \end{array}$$

where $S_{i}={\left[s_{1i},s_{2i},\cdots ,s_{ni}\right]}^{T}$ and $S={\left[S_{1},S_{2},\cdots ,S_{n}\right]}^{T}$.

According to the object function (20),

(21) $$E\left({\left\|S-S^{pw}\right\|}_{F}^{2}\right)=E\left(\sum_{i=1}^{n}\sum_{j=1}^{n}{\left(s_{ij}-s_{ij}^{pw}\right)}^{2}\right)=\sum_{i=1}^{n}\sum_{j=1}^{n}E{\left(s_{ij}-s_{ij}^{pw}\right)}^{2}$$

On the other hand, according to (19), we have

(22) $$s_{ij}-s_{ij}^{pw}=s_{ij}-\frac{1}{n}-\frac{1}{2\alpha }\left(\frac{\sum_{s=1}^{n}d_{is}^{pw}}{n}-d_{ij}^{pw}\right).$$

Therefore,

(23) $$E{\left(s_{ij}-s_{ij}^{pw}\right)}^{2}=E{\left(s_{ij}-\frac{1}{n}-\frac{1}{2\alpha }\left(\frac{\sum_{s=1}^{n}d_{is}^{pw}}{n}-d_{ij}^{pw}\right)\right)}^{2}$$

According to the properties of expectation and variance:

(24) $$E\left(X^{2}\right)=E^{2}\left(X\right)+D\left(X\right),$$

(25) E(aX+b)=aE(X)+b

and

(26) $$D\left(aX+b\right)=a^{2}\cdot D\left(X\right),$$

Equation (23) can be reduced to:

(27) $$E{\left(s_{ij}-s_{ij}^{pw}\right)}^{2}={\left(s_{ij}-\frac{1}{n}-\frac{1}{2\alpha }\left(\frac{\sum_{s=1}^{n}E\left(d_{is}^{pw}\right)}{n}-E\left(d_{ij}^{pw}\right)\right)\right)}^{2}+\frac{1}{4\alpha^{2}}D\left(\frac{\sum_{s=1}^{n}d_{is}^{pw}}{n}-d_{ij}^{pw}\right)$$

Obviously, (7) is equivalent to the following optimal model:

(28) $$\min{\sum_{i=1}^{n}\sum_{j=1}^{n}\left(s_{ij}-\frac{1}{n}-\frac{1}{2\alpha }\left(\frac{\sum_{s=1}^{n}E\left(d_{is}^{pw}\right)}{n}-E\left(d_{ij}^{pw}\right)\right)\right)}^{2}.$$

The optimal solution for the above optimal model can be obtained easily, which is

(29) $$s_{ij}=\frac{1}{n}+\frac{1}{2\alpha }\left(\frac{\sum_{s=1}^{n}E\left(d_{is}^{pw}\right)}{n}-E\left(d_{ij}^{pw}\right)\right),i=1,2,\cdots ,n.$$

Now, another understanding for a possible world is presented. Let us review the definition of possible world. The construction of an uncertain dataset and its PDF *f*(*pw*) are known. Then, if the dimensions of the dataset are finite, which is assumed to be ${\left\{o_{ij}\right\}}_{j=1}^{n}$, the edge probability density function (EPDF) for *i*th dimension is:

(30) $$f_{i}\left(O_{i}\right)=\int_{D_{pw}/D_{i}}f\left(pw\right)d\left(pw/O_{i}\right)$$

Moreover, if the dimensions of *O_i_*(*i* = 1, 2, …, *n*) are finite, which is assumed to be ${\left\{o_{ij}\right\}}_{j=1}^{n}$, the edge probability density function (EPDF) for *j*th dimension of *O_i_*(*i* = 1, 2, …, *n*) is:

(31) $$f_{ij}\left(o_{ij}\right)=\int_{D_{i}/D_{ij}}f_{i}\left(O_{i}\right)d\left(O_{i}/o_{ij}\right)$$

Here, it is assumed that *distance*(*O_i_*,*O_j_*) is the distance between the random variables *O_i_* and *O_j_*. Then, the consensus affinity matrix *S* can be obtained according to the following formula:

(32) $$\begin{array}{l} \min\sum_{j=1}^{n}d_{ij}s_{ij}+\alpha \sum_{j=1}^{n}s_{ij}\\ s.t.\;S_{i}={\left[s_{1i},s_{2i},\cdots ,s_{ni}\right]}^{T}\\ {\left(S_{i}\right)}^{T}\cdot 1^{n\times 1}=1\\ 0\leq s_{ij}\leq 1 \end{array}$$

where *d_ij_* = *g*(*distance*(*O_i_*,*O_j_*)), and $S={\left[S_{1},S_{2},\cdots ,S_{n}\right]}^{T}$.

Then, according to the analysis above, if there is no extra information about classes, the optimal solution for the object function (15) is:

(33) $$s_{ij}=\frac{1}{n}+\frac{1}{2\alpha }\left(\frac{\sum_{s=1}^{n}d_{is}}{n}-d_{ij}\right).$$

Compared with (12), the distribution is used instead of the expectation of point distance. Therefore, (12) is appropriate for the possible world that includes fewer and simpler random variables, while (16) is appropriate for the possible world with complexity random variables in theory.

So far, when the distance-based function is confirmed, the optimization consensus affinity matrix *S* for the all possible worlds can be worked out. 

According to the calculations above, the closer two data objects are, the larger *s_ij_* is. Therefore, the value of *s_ij_* may have no use when *s_ij_* < *p* (distance threshold). Then, the matrix S may need to be pruned to remove the meaningless *s_ij_*. This pruning is divided into two steps: removing and normalization. In the removing step, the meaningless values are replaced by 0. In the normalization step, the meaningful value is recalculated to keep the equation:

(34) $$\sum_{i=1}^{n}s_{ij}=1,j=1,2,\cdots ,n.$$

The following Algorithm 1 shows the processing of pruning:
**Algorithm 1** for Matrix Pruning:Input: the matrix *S* ∈ *R*^*n*×*n*^ and pruning threshold *p* The processing: Removing step: For *i* = 1 to *n* For *j* = 1 to *n* If *s_ij_* < *p* *s_ij_* = 0 End if End for End for Normalization step: For *i* = 1 to *n* $sum_{i}=\sum_{j=1}^{n}s_{ji}$ For *j* = 1 to *n* $s_{ji}=\frac{s_{ji}}{sum_{i}}$ End for End for

Moreover, in spectral analysis, if a nonnegative affinity matrix *S* is given, the corresponding Laplacian matrix *L_s_* can be calculated as $L_{s}=D_{s}-\frac{S^{T}+S}{2}$, where *D_s_* is a diagonal matrix and its *i*th diagonal element is $\sum_{j=1}^{n}\frac{s_{ij}+s_{ji}}{2}$. The Laplacian matrix *L_s_* has an important property as follows [24].

**Theorem** **1.** *Let S be a nonnegative affinity matrix; then, the multiplicity k of the eigenvalue 0 of the Laplacian matrix L_s_ is equal to the number of connected components in the graph associated with the affinity matrix S.*

*It is assumed that the eigenvalues of the Laplacian matrix L_s_, which is ${\left\{\sigma_{i}\right\}}_{i=1}^{n}$, are ordered from small to large. According to the properties of the Laplacian matrix L_s_, we have the following conclusion:*

(35) $$0=\sigma_{1}\leq \sigma_{2}\leq \cdots \leq \sigma_{n}.$$

*If the number of clusters k is unknown, the threshold Th is set to decide k, which satisfies:*

(36) $$\sigma_{k}\leq Th\leq \sigma_{k+1}.$$

Finally, the eigenvectors of eigenvalues *σ*_1_ to *σ_k_* comprise the matrix *U* ∈ *R^n×k^*. The k-means clustering algorithm is used to cluster the row of matrix *U*. The clustering result is that of the training set. The Algorithm 2 for processing *S* is shown as follows.
**Algorithm 2** for processing S:Input: the matrix *S* ∈ *R^n×n^* and clustering threshold *Th* The processing:
$L_{s}=D_{s}-\frac{S^{T}+S}{2}$ ${\left\{\sigma_{i}\right\}}_{i=1}^{n}$ is the set of eigenvalues of *L_s_* $0=\sigma_{1}\leq \sigma_{2}\leq \cdots \leq \sigma_{n}.$ ${\left\{\upsilon_{i}\right\}}_{i=1}^{n}$ is the set of eigenvectors of *L_s_* If $\sigma_{r}\leq Th\leq \sigma_{r+1}$ *k* = *r* End if $U=\left[\upsilon_{1},\upsilon_{2},\cdots ,\upsilon_{k}\right]$

Cluster the row of matrix U according to the k-means method. These are also the clustering results for training set. Therefore, the cluster ${\left\{C_{i}\right\}}_{i=1}^{k}$, and the number of cluster members ${\left\{n_{i}\right\}}_{i=1}^{k}$ are obtained.

### 4.4. Updating

After clustering the training set, the data in the test set should be put into the clusters determined above. Firstly, the test set is given as follows:

The Test Set: $O={\left\{O_{i}\right\}}_{i=n}^{n+p}$, and $O_{i}={\left[o_{1i},o_{2i},\cdots ,o_{ni}\right]}^{T}$ is the data object.

The clustering updating algorithm for the test set is divided into two steps: clustering and updating. The details are shown in the following Algorithm 3:
**Algorithm 3** for Clustering Updating:Input: the center of each cluster ${\left\{C_{i}\right\}}_{i=1}^{k}$, and the number of cluster members ${\left\{n_{i}\right\}}_{i=1}^{k}$ of training set and the test set $O={\left\{O_{i}\right\}}_{i=n}^{n+p}$.
The processing:
Clustering step:
${\left\{C_{i}^{\prime }\right\}}_{i=1}^{k}$ = ${\left\{C_{i}\right\}}_{i=1}^{k}$.
For *i* = *n* + 1 to *n* + *p* ${\left[d_{ij}\right]}_{j=1}^{k},d_{ij}=distance\left(O_{i},C_{j}\right)$ ${\left[d_{ij}^{\prime }\right]}_{j=1}^{k},d_{ij}=distance\left(O_{i},C_{j}^{\prime }\right)$ $cluster_{i}=\underset{j}{\arg\min}\;d_{ij}$
${cluster}_{i}^{\prime }=\underset{j}{\arg\min}\;d_{ij}^{\prime }$ If *cluster_i_* = *cluster_i_*’ *O_i_* belongs to *cluster_i_*. Else if $\frac{d_{i,cluster_{i}}}{d_{{i,cluster}_{i}^{\prime }}}\geq \frac{d_{i,cluster_{i}^{\prime }}^{\prime }}{d_{i,cluster_{i}}^{\prime }}$ *O_i_* belongs to *cluster_i_*’ Else *O_i_* belongs to *cluster_i_* End if End if End for Centers updating step: For *i* = *n* + 1 to *n* + *p* If *O_i_* belongs to *cluster_i_* $C_{cluster_{i}}^{\prime }=\frac{n_{cluster_{i}}C_{cluster_{i}}^{\prime }+O_{i}}{n_{cluster_{i}}+1}$ $n_{cluster_{i}}=n_{cluster_{i}}+1$ End if End for

## 5. Simulations

In this section, comparisons with three state-of-the-art uncertain data clustering algorithms are conducted on real benchmark datasets. Moreover, an uncertain dataset that obeys the multivariate Gaussian distribution is generated, and the parameters in the PWFEM model are discussed.

In the comparisons, six common real benchmark datasets, which came from ‘http://archive.ics.uci.edu/ml/’, are employed for the simulation; their details are shown in Table 1:

These datasets were originally established as collections of data with determinate values. Then, we followed the method in [27] to generate uncertainty in these datasets, and the generation method is shown as follows Algorithm 4:
**Algorithm 4** The Generation Method from Numerical Data to Uncertain Data (Gaussian Type).Input: the numerical data $a={\left[a_{1},a_{2},\cdots ,a_{n}\right]}^{T}$ and the standard deviation of each attribute $\left[\sigma_{1},\sigma_{2},\cdots ,\sigma_{n}\right]$ Output: the corresponding uncertain data $ua={\left[ua_{1},ua_{2},\cdots ,ua_{n}\right]}^{T}$ For *i* = 1 to *n* *x* = random, 0 < *x* ≤ 1 $ua_{i}=\frac{1}{\sqrt{2\pi }\sigma }e^{-\frac{{\left(x-a_{i}\right)}^{2}}{2\sigma^{2}}}$ End for

### 5.1. The Clustering Accuracy

In this part, 2 widely used evaluation metric, which are accuracy (ACC) and Normalized mutual information (NMI), are adopted to compare the different clustering algorithms. In this part, the proposed clustering algorithms, PWFEM-nd and PWFEM-pd, are compared with three state-of-the-art uncertain data clustering algorithms: UK-means [26], REP [27] and PWCLU. Each clustering algorithm was run 100 times. The maximum, minimum, mean value, and variance of the ACC were calculated with respect to each algorithm. The comparisons were simulated for two cases. Case 1 is the real mean value with variance known, while case 2 is the finite measurement results, which obey the given PDF instead.

In order for the proposed model to be executed properly, the exact values of expectation and covariance need to be known. However, the datasets used in this simulation do not give those values. Therefore, the approximate values were calculated instead according to the following formula:

(37) E=X and Cov=Cov(X).

where $X={\left\{x_{i}\right\}}_{i=1}^{n}$ is the dataset.

The comparisons of ACC for each algorithm in case 1 are shown in Table 2.

As shown in Table 2, in the datasets of wine and glass, the PWFEM-nd shows the best performance with maximum, minimum, and mean values. Unfortunately, it shows the worst performances with those values in the datasets of iris, Ecoli and PhishingData. As for the proposed PWFEM-pd, it shows the best performances with maximums in all datasets except wine and glass.

According to their respective algorithms, there may be plenty of reasons for the results above. Some analyses that have high probabilities are presented next.

Firstly, it is important to note that the UK-means, REP, PWCLU, and PWFEM-nd use the mean value only. Therefore, their variance values are zeros, which means the clustering results never change throughout the 100 iterations. Only PWFEM-pd uses the variance of uncertain data.

Secondly, UK-means clusters the dataset directly, while REP, PWCLU, PWFEM-nd, and PWFEM-pd cluster the dataset indirectly. Here, REP, PWCLU, PWFEM-nd, and PWFEM-pd use the model based on the possible world. Moreover, PWCLU uses the Euclidean distance (‖∙‖2). PWFEM-nd uses the cosine similarity. PWFEM-pd uses the Kullback–Leibler divergence. Compared with the PWCLU, PWFEM-nd combines the distributions of each component in a datum. Moreover, PWFEM-pd calculates the distance in distributions directly, while PWCLU and PWFEM-nd transform the distributions into some special numbers (mean value and variance). Therefore, the clustering accuracy of PWFEM-pd may be higher than that of PWCLU in most cases. Moreover, PWFEM-pd can be regarded as having different covariances obtained randomly to that of clustering. If a covariance close to the true covariance is acquired, a high accuracy of clustering is gained. 

**Figure 1:**
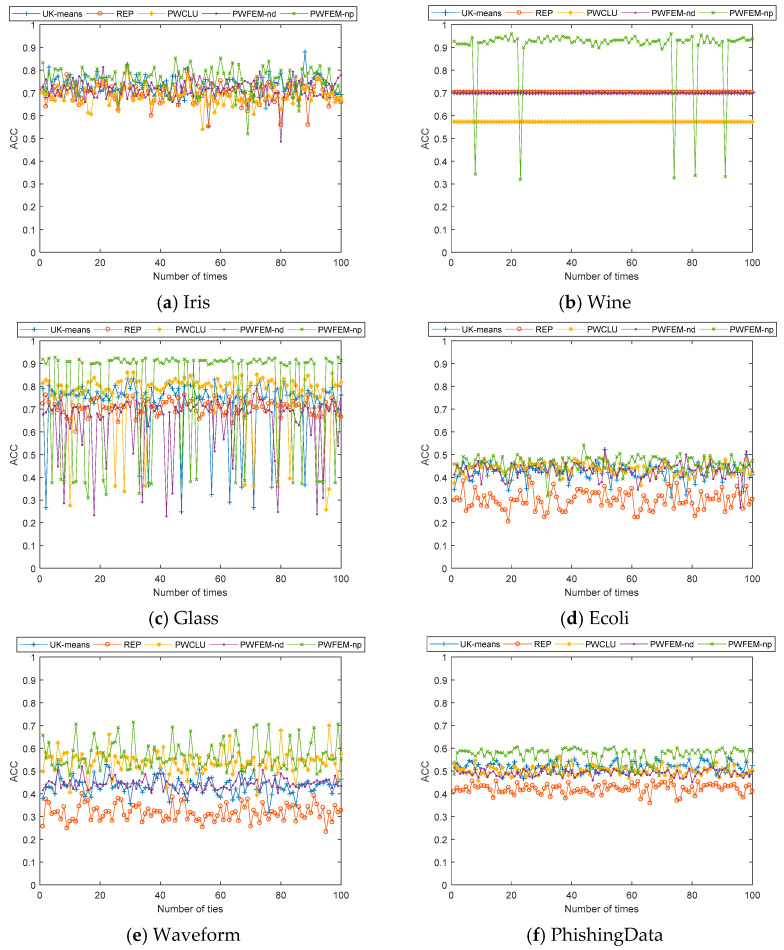
ACC with different clustering algorithms for 100 iterations in case 1.

For a clearer view of the changing of clustering accuracy with different covariances, see Figure 1.

As shown in Figure 1, the ACC of PWFEM-pd is sensitive to the covariance of the uncertain data. On the other hand, the impacts caused by covariances from different datasets lead to different results. Obviously, in Figure 1a,c,d,f, the ACC is highly dependent on the covariance. In Figure 1e, the ACC is divided into two parts: one is around 0.51 and the other is around 0.34, when different covariances are given. Moreover, in Figure 1b, the ACC is stable around 0.5 most times with the changing of covariance.

**Figure 2:**
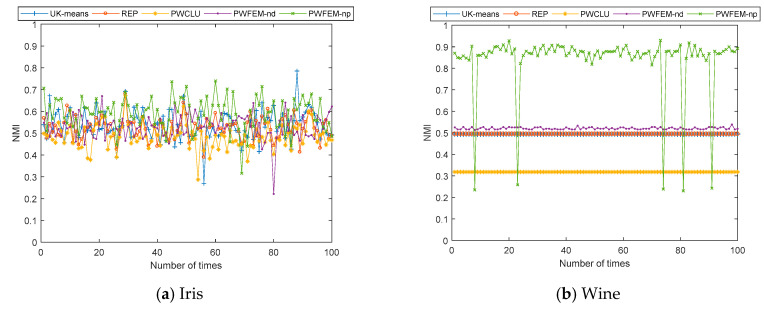

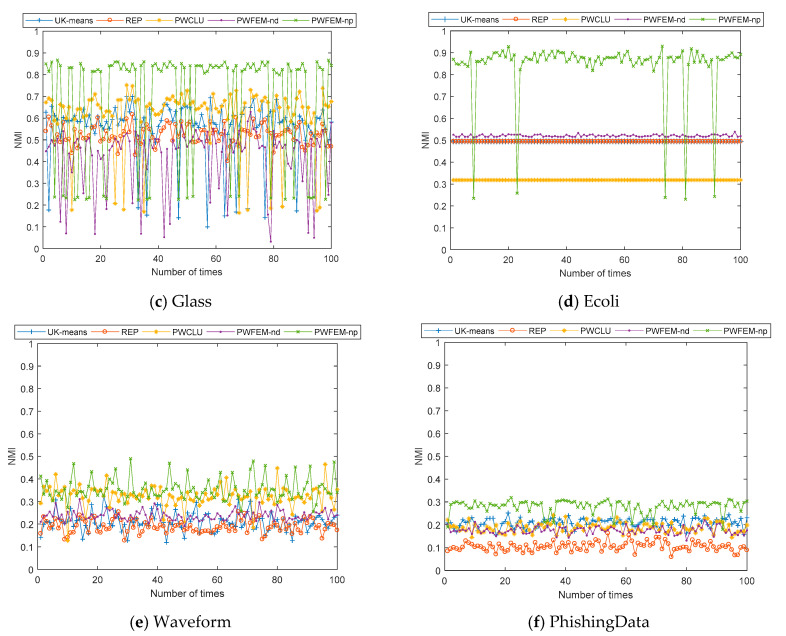
NMI with different clustering algorithms for 100 iterations in case 2.

According to the analysis above, only for the proposed models, which are PWFEM-nd and PWFEM-pd, the ACC is sensitive to covariance. Then, the changing of mean values is added to the simulations. Therefore, the simulation results are given for case 2, which uses the generation method proposed in the beginning of this section; the results of case 2 are shown in Table 3 and Figure 2.

As shown in Table 3, when combining the maximum value and minimum value, the clustering results of all clustering methods change. This means all the clustering methods are sensitive to the mean value. Moreover, the sensitivity to each clustering method varies. Obviously, the fluctuation ranges of all clustering methods in iris and glass are the most drastic. On the other hand, the clustering accuracy of the PWFEM-pd algorithm is always higher than that of the PWFEM-nd, but its stability is lower than that of the PWFEM-nd. Besides, compared with Table 2, the NMI are lower than the ACC for the same dataset, which means that in the clustering results of the model, the accuracy of each class is inconsistent, with some categories having high precision and some having low precision.

For a clearer view of the changing of clustering accuracy with different covariances and mean values, see Figure 2.

As shown in Figure 2, the PWFEM-pd has a similar fluctuation as that shown in Figure 1. Unfortunately, this clustering method is sensitive to both mean value and covariance. Therefore, it is hard to distinguish the main reason. Next, the remaining four clustering methods are discussed.

Firstly, similar to the conclusion in Table 2, Figure 2c,d in all clustering methods show a drastic fluctuation. For UK-means and CK-means, they show a drastic fluctuation in Figure 2a and are stable in Figure 2b,e,f. For PWCLU, it is stable in Figure 2a,b,e,f. For PWFEM-nd, it is stable in Figure 2a,b,f, while it is stable at two ranges in Figure 2e.

According to the analysis above, the situations of the proposed methods are clearer. However, the variation tendency with the mean value and covariance are not clear. Therefore, a specific dataset was generated to investigate the above issues.

### 5.2. The Simulation with a Specific Dataset

In this part, a specific dataset is generated to analyze the impacts of mean value and covariance. The generated dataset consisted of two dimensions, and the number of data points was set at 1000. It was divided into three clusters, whose centers were [0, 0], [100, 0], and [0, 100]. The distance between the datum and its center was randomly distributed in [0, *r*]. The variance for each dimension was *σ_i_*(*i* = 1, 2). Moreover, it was set as *σ*_1_ = *σ*_2_ = *σ*. The correlation coefficient of these two dimensions was *ρ*. Therefore, the covariance of this dataset was:

$$\left[\begin{array}{cc} \sigma & \rho \sigma \\ \rho \sigma & \sigma \end{array}\right].$$

Next, the parameters *r*, *σ,* and *ρ* are discussed.

**Figure 3:**
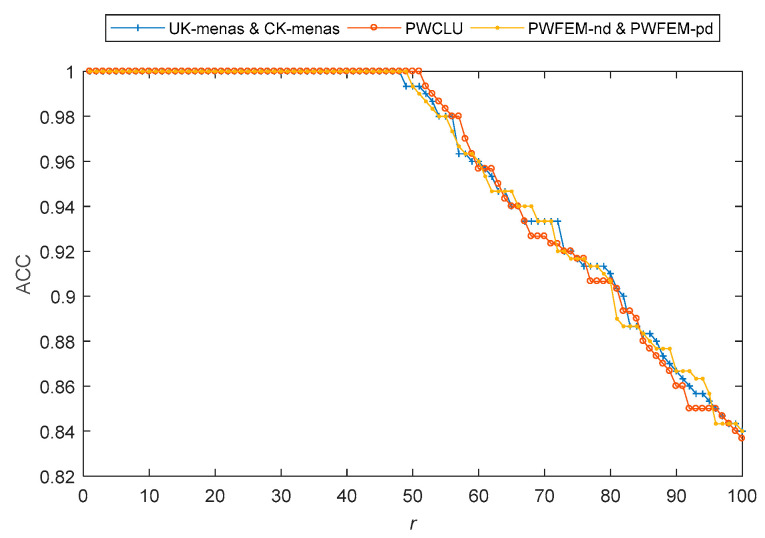
Change of ACCs with *r* values from 1 to 100.

In this simulation, *σ* = 2, *ρ* = 0, and *r* was from 1 to 100. As shown in Figure 3, the ACCs of all methods were 1 before about 50, and then reduced with increasing *r*. This simulation results are in accordance with common sense.

On the other hand, if *ρ* = 0 and *r* is fixed, *σ* can vary the distance between the data evenly. Therefore, it cannot affect the clustering results, and the simulation proves it. Because the ACC curves of all methods are lines parallel to the *X*-axis, the figure was omitted.

**Figure 4:**
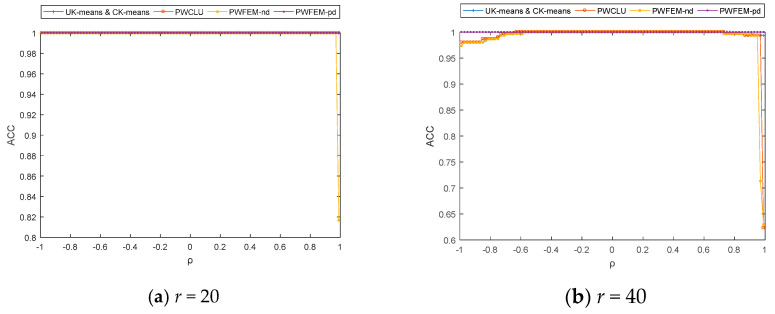

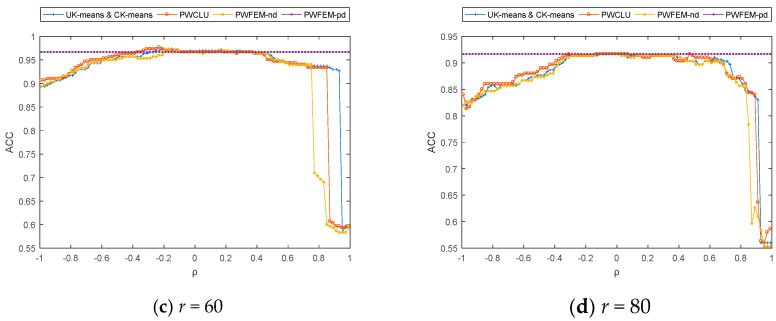
Change of ACCs with *ρ* from −1 to 1.

Finally, the simulation for *ρ* is discussed with *σ* = 2, *r* = 20, 40, 60, and 80, and −1 < *ρ* < 1. The simulation results are shown in Figure 4.

As shown in Figure 4, when *r* < 50, the ACCs are stable for all methods with −1 < *ρ* < 1. This is because the cluster structure is prominent in this condition, whereas the effect of ρ on the clustering result is weak. Moreover, when *r* > 50, the ACCs of UK-means, CK-means, PWCLU, and PWFEM-nd show significant changes between in (−1, −0.7) and in (0.7, 1). In these two intervals, *ρ* makes the data points even messier. Therefore, the ACCs of clustering results decrease if the data points are not processed. On the other hand, the ACCs become stable when −0.7 < *ρ* < 0.7. Obviously, the effect of *ρ* on the clustering results is weak.

## 6. Conclusions

In this paper, a possible world-based fusion estimation model for uncertain data is proposed. It includes two methods, which are the PWFEM-nd and PWFEM-pd. The PWFEM-nd is based on a data perspective, which uses a bottom-up method to cluster the data. The PWFEM-pd uses clustering according to the uncertain data directly. Both these methods depend more on the probability density distribution of uncertain data. We performed some simulations and confirmed that the proposed methods showed better performance in terms of probabilistic accuracy. The accuracy is highly dependent on the accuracy of covariance.

The discussion in the last section is incomplete. Obviously, it gets more complex when dimension increases. Only some simple conclusions are given in the simulation. In addition, the exact covariance is not usually obtained in actual scenarios. In any case, the proposed methods provide a new way to treat uncertain data clustering. The issues mentioned above are also to be addressed in future works.

## Figures and Tables

**Table 1:** Details of the adoptive datasets [25].

Dataset	Objects	Attributes	Classes
Iris	150	4	3
Wine	178	13	3
Glass	214	9	6
Ecoli	327	7	5
Waveform	5000	21	3
PhishingData [26]	1353	9	3

**Table 2:** Accuracy (ACC) for each algorithm in case 1.

		UK-Means	REP	PWCLU	PWFEM-nd	PWFEM-pd
Iris	Max	0.8800	0.8133	0.8133	0.8133	0.8533
	Min	0.5533	0.5533	0.5400	0.4867	0.5200
	Mean	0.7244	0.6994	0.6869	0.7181	0.7602
	Variance	0.0022	0.0021	0.0016	0.0017	0.0028
Wine	Max	0.7022	0.7022	0.5730	0.7079	0.9607
	Min	0.7022	0.7022	0.5730	0.6966	0.3202
	Mean	0.7022	0.7022	0.5730	0.6989	0.8999
	Variance	0	0	0	0	0.0173
Glass	Max	0.8333	0.7619	0.8618	0.7905	0.9286
	Min	0.2476	0.6000	0.2571	0.2286	0.3095
	Mean	0.7239	0.7078	0.7588	0.6489	0.7818
	Variance	0.0191	0.0010	0.0204	0.0173	0.0537
Ecoli	Max	0.5327	0.4953	0.5374	0.5234	0.5421
	Min	0.3458	0.2056	0.4065	0.3318	0.4299
	Mean	0.4422	0.4025	0.4905	0.4527	0.4634
	Variance	0.0012	0.0035	0.0011	0.0014	0.0009
Waveform	Max	0.5291	0.4006	0.7003	0.5199	0.7156
	Min	0.3180	0.2324	0.3945	0.4006	0.4801
	Mean	0.4350	0.3177	0.5403	0.4445	0.5706
	Variance	0.0014	0.0013	0.0025	0.0006	0.0038
PhishingData	Max	0.5639	0.4560	0.5647	0.5188	0.6061
	Min	0.4664	0.3585	0.4568	0.4508	0.4797
	Mean	0.5183	0.4218	0.5027	0.4910	0.5719
	Variance	0.0005	0.0004	0.0004	0.0002	0.0010

**Table 3:** NMI for each algorithm.

		UK-Means	REP	PWCLU	PWFEM-nd	PWFEM-pd
Iris	Max	0.7854	0.6809	0.6716	0.6700	0.7396
	Min	0.2694	0.3898	0.2871	0.2213	0.3162
	Mean	0.5374	0.5245	0.4834	0.5295	0.5927
	Variance	0.0050	0.0027	0.0031	0.0033	0.0054
Wine	Max	0.4946	0.4946	0.3184	0.5389	0.9551
	Min	0.4946	0.4946	0.3184	0.5136	0.3146
	Mean	0.4946	0.4946	0.3184	0.5209	0.8803
	Variance	0	0	0	0	0.0198
Glass	Max	0.7001	0.6171	0.7522	0.6288	0.8671
	Min	0.0997	0.4028	0.1643	0.0320	0.2233
	Mean	0.5511	0.5250	0.6094	0.4258	0.6911
	Variance	0.0196	0.0019	0.0223	0.0204	0.0672
Ecoli	Max	0.6544	0.6544	0.7064	0.7125	0.7309
	Min	0.3731	0.3731	0.5199	0.4679	0.3639
	Mean	0.4988	0.4988	0.6354	0.5629	0.5569
	Variance	0.0050	0.0050	0.0034	0.0054	0.0079
Waveform	Max	0.3247	0.2548	0.4645	0.3104	0.4895
	Min	0.1195	0.1286	0.1282	0.1919	0.2545
	Mean	0.2112	0.1909	0.3244	0.2392	0.3558
	Variance	0.0017	0.0008	0.0022	0.0005	0.0025
PhishingData	Max	0.2517	0.1636	0.2416	0.2200	0.3190
	Min	0.1559	0.0594	0.1452	0.1313	0.1804
	Mean	0.2088	0.1050	0.1880	0.1760	0.2803
	Variance	0.0004	0.0004	0.0005	0.0003	0.0008

## Data Availability

The data presented in this study are openly available in “http://archive.ics.uci.edu/ml/index.php”, reference numbers are [25,26].
